# Pain Management and Analgesics Used in Small Mammals during Post-Operative Period with an Emphasis on Metamizole (Dipyrone) as an Alternative Medication

**DOI:** 10.3390/molecules27217434

**Published:** 2022-11-01

**Authors:** Georgiana Lupu, Lucia Bel, Sanda Andrei

**Affiliations:** 1 Department of Preclinical Sciences, Faculty of Veterinary Medicine, University of Agricultural Sciences and Veterinary Medicine Cluj-Napoca, 400372 Cluj-Napoca, Romania; 2 Department of Paraclinical and Clinical Sciences, University of Agricultural Sciences and Veterinary Medicine Cluj-Napoca, 400372 Cluj-Napoca, Romania

**Keywords:** metamizole, postoperative pain management, small mammals, analgesia

## Abstract

Metamizole (dipyrone) is a widely used non-opioid analgesic in both human and animal medicine. Metamizole’s safety has been the topic of numerous opposing debates, given the fact that in certain countries metamizole is frequently used as an over-the-counter (OTC) medicine, while in others it is banned due to the risk of agranulocytosis. Further, small mammals such as rabbits, ferrets, rodents, and hedgehogs have become some of the most common pets present in veterinary practice, and each of these species has specific analgesic needs due to their anatomy and physiology. The key to providing appropriate medical care is in finding a substance that has minimal negative effects. In small mammals, analgesia is an important factor and, it happens frequently that, pain in these patients is not well managed. Post-operative pain management is an important topic in the welfare of animals. The objectives of this review, thus, were to provide a concise overview of analgesics that are used in the treatment of postoperative pain in small mammals (e.g., rabbits and rodents) and to highlight the importance of this product, metamizole, in veterinary medicine, as well as the potential of this substance as an alternative analgesic for the treatment of postoperative pain in small mammals.

## 1. Introduction

Nowadays, small mammals such as rabbits, ferrets, rodents, and hedgehogs have become some of the most common pets present in veterinary practice. Establishment of new standards of therapy for these species, however, is required. The anesthetic protocols for these species are poorly understood and are often extrapolated from protocols for other well-known species, including dogs and cats. Small mammals are also known for the difficulties in conducting a thorough clinical examination of them safely. They are extremely vulnerable to stress and incorrect handling can cause harm to them. Sedation of the animal is therefore required for some procedures, in order to minimize negative side effects (in the case of a procedure that could cause the animal stress and pain).

Small mammals are presented at the clinic for a multitude of therapeutic protocols and surgical interventions. An appropriate analgesic and anesthetic protocol is essential in the veterinary clinic in order to perform these treatments safely.

The pharmacokinetic activity of metamizole and its metabolites in other species, particularly in exotic animals (NAC), is not well known. The development of particular protocols can be made possible by obtaining accurate information and statistics on these patients, who are becoming more commonly treated in veterinary practice.

Post-operative pain management is an important topic in the welfare of animals. The primary objective of this study was to provide a concise overview of the analgesics used in the treatment of postoperative pain in small mammals (e.g., rabbits and rodents). In the veterinary medical market, metamizole is approved for use in other species, such as in swine, cattle, equines, and canines. The second objective was to present the current literature regarding the use of metamizole, in some animals and humans, with reference to its specific metabolism and mechanism of action. By reviewing this data, our aim was to highlight the importance of this product in veterinary medicine and the potential of this substance as an alternative analgesic for the treatment of postoperative pain in small mammals.

## 2. Pain Management

Analgesia is defined as the absence of sensitivity to pain, in particular the relief of the patient from pain without the loss of consciousness [1]. Analgesia is an essential aspect in exotic animal care, and the pain in these patients can often be left undertreated [2].

A study carried out with the help of veterinarians showed that the administration of substances with analgesic properties is less common in small mammals than in dogs and cats. For example, postoperative analgesia was administered in a percentage of 50–70% in dogs and cats, compared to 21% in rabbits and rodents. This may also be due to the difficulty in assessing the degree of pain experienced by the animal, but also the lack of information on the efficacy and safety of the substances used in these species [3,4,5].

A survey was conducted with practicing veterinarians in 2020 about how they can assess and relieve pain in rabbits. The survey showed that 94.3% of respondents routinely administered non-steroidal anti-inflammatory drugs to rabbits after surgery (e.g., spaying and neutering), 71.4% administered an opioid and 70.3% had administered a multimodal analgesia protocol. Buprenorphine and meloxicam were the most commonly used substances with analgesic properties. The study concluded that analgesia administered to rabbits has improved in recent years. However, the lack of a specific pain monitoring scale for them makes it difficult to monitor pain in this species [6]. Since a typical behavior does not always imply a pain-free state, determining if an animal is in pain is, therefore, more challenging. Despite the painful sensation, the animal may show “normal” behavior as an intrinsic response to avoid predators. As a prey species, the clinical manifestation of pain is more subtle due to the fact that they can hide pain symptoms very well. Most species adopt an immobile posture when in contact with the examiner, especially rabbits and guinea pigs [7,8,9].

Parameters used to measure response to painful stimuli in rodents are categorized into three main categories: physiological, biochemical, and behavioral. Indirect methods to measure pain include cardiac frequency and respiratory rates. In either case, handling the animal, stress, or excitement will increase both heart and respiratory rate. Biochemical parameters, including corticosteroid levels, catecholamines, and various hormones are commonly used in determining and quantifying pain. However, behavioral changes are often the most observed [1,10].

To quantify pain, the majority of pain scoring systems use one or a combination of instruments. The composite pain scale is an efficient and practical tool for the clinician in the immediate and structured measurement of pain in animals of different species, such as dogs and cats. However, in rabbits and rodents there is not yet an accepted pain scale equivalent. The only way of quantifying pain in this species is by recording the Grimace Scale (RbGS), which uses changes in facial expression [11].

RbGS was created by adapting pain assessment methods from rodents, such as mice, which was developed by Langford [12] and Sotocinal [13]. The RbGS is composed of five indicators that are represented by facial expression changes (FAU), such as the: narrowing of the eye socket; flattening of the cheek; change in the shape of the nostrils; change in the position of the whiskers, and change in the position of the ears [14]. RbGS should only be used in animals who have fully recovered from anesthesia. To avoid changes in facial expression that are unrelated to the animal’s condition, the animal must be monitored for a brief period of time. The examiner rates the facial alterations on a scale of 0 to 2 (0 = no pain, 1 = mild presence of pain, and 2 = apparent presence of pain). RbGS score is the sum of the values assigned to each change [14].

A novel composite pain scale called CANCRS, developed by Banchi et al., 2020, which combines RbGS with a range of clinical parameters (CPS), has been created and tested on various breeds of rabbits. The CPS includes physiological parameters (pupil dilation, respiratory rate, heart rate, and breathing pattern) and behavioral responses (response to palpation, metal status, and vocalizations). For each parameter, a score was assigned according to pain intensity, and at the end the total sum of the scores was calculated. The scores were equally distributed into four pain classes represented by: no pain (NP), discomfort (D), moderate pain (MP), and severe pain (SP) [11]. 

## 3. Drugs Used in Small Mammal Analgesia Protocols

There are various classes of anesthetic drugs available, and the effects that have been shown so far may vary according to the species (they also differ between individuals of the same species). A multimodal anesthetic protocol, which includes compounds from several distinct classes, offers the chance to achieve a more balanced anesthesia—for example by including analgesia when necessary—in spite of the clinician’s temptation to use only one drug in order to simplify the anesthetic protocol. The doses of the substances will automatically be lower with fewer side effects if a multimodal anesthesia strategy is adopted [15]. 

*Fentanyl* is an opioid agonist that acts primarily on µ-receptors and is considered 50–100 times more potent than morphine, with a rapid onset of effect and a short duration of action [16]. Due to its limited bioavailability, fentanyl is ineffective when administered orally; however, it can be administered intravenously, as a bolus or infusion, subcutaneously, or intramuscularly. Duration of effect depends on the route of administration and dosage, and can range from 30 min to 2 h [17]. It is an excellent painkiller for moderate somatic and visceral pain [16].

*Buprenorphine* is a partial µ-agonist opioid agent that provides potent analgesia that is maintained for 6–8 h [18]. In rabbits, it is used to provide a long-lasting, analgesic effect and is also used post-operatively for the treatment of moderate to severe pain [1]. Installation of analgesia is quick—i.e., 30 min after intravenous administration. Buprenorphine has been shown to have an effective absorption through the oral mucosa, thereby making it a good method for the purposes of giving treatment at home, by the owners [2]. Reduced gastrointestinal motility is one of buprenorphine’s frequent side effects. However, it has not been demonstrated to be clinically significant as, in most cases, persistent, unrelieved pain in rabbits results in intestinal stasis syndrome [2].

*Butorphanol* is a synthetic opioid with mixed k receptor agonist/antagonist properties on µ receptors, which tends to have higher bioavailability and faster elimination in many animal species. Compared to other opioids, the effects on the respiratory system are less significant. Butorphanol provides good analgesia for visceral pain, but has a limiting effect on somatic pain [16]. Since the action lasts about 2–4 h, regular dosing is required to maintain analgesia. Additionally, it has sedative effects and is frequently used as a preventative analgesic for the purposes of preoperative medication [8].

*Tramadol* is a weak opioid with some analgesic effects. Mice, rats, and leporids have been used in studies on the pharmacokinetics of this substance. Although studies testing its effectiveness in mice have revealed varying results, its oral bioavailability makes it appropriate for postoperative pain control. Tramadol undergoes rapid metabolism into its metabolites, most of which have analgesic properties in most species. In rats, the primary metabolite of tramadol is more potent than tramadol itself. In rabbits, the plasma concentration of tramadol was at a low level after oral administration, and doses used for analgesic effect (4.4 mg/kg IV) had minimal effect on the alveolar concentration of isoflurane. Until clinical efficacy in rabbits is demonstrated, this substance should only be used if other therapies have been found to be ineffective [9].

*NSAIDs* have an anti-inflammatory, analgesic, and antipyretic effect. It has been demonstrated that when opioids are used in combination, a smaller amount of opioid is sufficient as the cumulative effects are more effective [8]. Meloxicam, carprofen, and ketoprofen are the *NSAIDs* that are administered to rabbits and rodents most commonly. According to pharmacokinetic studies, rabbits metabolize meloxicam more quickly than rodents, dogs, and even humans, and have a lower peak plasma concentration of the drug after oral treatment. In rabbits, an initial dose of 1 mg/kg followed by 0.5 mg/kg/day is sufficient in order to provide adequate postoperative analgesia [2].

Intraoperative analgesia is provided by techniques that use local and regional anesthetics. The most popular medications are lidocaine and bupivacaine, which can be used topically, intravenously, intraarticularly, as regional nerve blocks, or epidurally [2]. Local anesthetics block sodium channels, preventing nerve impulse production and conduction. Local anesthetic can be used as an addition to general anesthesia in order to lower the amount of drug administered. It may also help to lower the requirement for postoperative analgesia [8].

Although there is sufficient data demonstrating the efficacy of *acetaminophen* in studies on pain in animals, the results offered in the use of this substance for postoperative pain have been disappointing. There have been only a few studies on postoperative pain, therefore it is difficult to say with certainty if acetaminophen combined with NSAIDs or opioids will be more beneficial. One positive aspect of acetaminophen is that it is found in the form of a pleasant tasting solution [9].

## 4. Metamizole

### 4.1. Current Controversies of Metamizole in Human Medicine

Metamizole, known as dipyrone, is a non-opioid analgesic and antipyretic with spasmolytic properties, which is used in human and veterinary medicine for peri- and post-operative pain control [19]. Metamizole use is currently a controversial topic in the field of human medicine. This is due to concerns regarding human safety; as a result, it has been withdrawn off the market in various countries [19]. Metamizole is regarded as one of the most potent and secure non-opioid analgesics, however there is evidence to suggest that extended treatment may result in a variety of adverse effects. These can range from damage to the hematological system—which lead to leukopenia—agranulocytosis, and occasionally even aplastic anemia in humans, though this was the most severe reaction noted [20]. According to other studies, the frequency of these reactions is quite low compared to the advantages offered. Metamizole is still a prescription medication, however it is also accessible over-the-counter (“OTC”) in pharmacies in some countries, including Brazil, Mexico, China, Switzerland, Germany, and Eastern Europe [21].

One of the most commonly prescribed painkillers in Germany is metamizole since it was first synthesized there in 1920 under the name “Novalgin”. The use of it is indicated in cases of intestinal colic, post-operative pain, cancer-related or trauma-related distress, as well as fever that does not diminish in response to conventional therapies. In the Netherlands, it is only registered for IV administration for the purposes of the treatment of severe acute pain or high grade fever, where other products are contraindicated. In Spain, metamizole has been one of the most widely used analgesics for treating postoperative pain in children. In Serbia, it is a licensed product for short-term use in post-traumatic pain and postoperative pain [21].

The controversy around metamizole’s safety originates from the fact that it is widely used as an over-the-counter medication in some countries, while also being restricted in others (due to the possibility of agranulocytosis). However, there is a small but fatal risk associated with metamizole, which includes agranulocytosis and anaphylactic shock. The onset of agranulocytosis is unpredictable and fatal cases have been reported. This is following intermittent use or short-term use of metamizole, as well as after long-term administration; as such, a hypersensitivity mechanism is speculated. In otherwise healthy individuals, metamizole seems to have no noticeable gastrointestinal side effects and has no impact on renal function. However, having said that, prolonged use could be harmful to the kidneys. In addition, allergies can also trigger respiratory problems and skin rashes [22].

The prevalence of metamizole-induced agranulocytosis varies geographically, as well as between studies. This is mostly caused by differences in the research approach, methodology, dosage, duration, and concurrent drug administration. However, some genetic predispositions could not be excluded, as metamizole is strongly associated with agranulocytosis in certain regions of the world, but for reasons that are, at this time, not fully understood [22].

### 4.2. Mechanism of Action

The mechanism of action for this substance is not yet fully known. Its effects may be due to suppression of prostaglandin synthesis via inhibition of iso-enzyme cyclooxygenase-3 and by the activation of the cannabinoid and opioidergic systems [23]. On the other hand, the anti-inflammatory effect of metamizole is low due its weak affinity to cyclooxygenase in environments that are rich in peroxidase, such as in inflamed tissues. It has also been reported that metamizole has a dose-dependent effect on platelet aggregation. It also possesses a spasmolytic effect on smooth muscle [21].

A study, showed in earlier research, demonstrated that metamizole causes competitive inhibition of cyclooxygenase (COX) [24]. Common anti-inflammatories have the ability to inhibit cyclooxygenases, which results in a reduction in inflammation, which causes analgesia as a side benefit. Several studies have demonstrated the inhibition activity of metamizole in vitro [25,26] and in vivo [27,28,29]. Metamizole metabolites—4-methylaminoantipyrine (4-MAA) and 4-aminoantipyrine (4-AA)—did not reduce COX activity in vitro as well as in standard COX inhibitors, but instead diverted the prostaglandin synthesis process, thereby preventing COX inhibition by attaching to its active site [29,30]. Metamizole is considered to be a COX-3 isoenzyme inhibitor, lowering prostaglandin synthesis in the dorsal horn of the spinal cord [26].

Metamizole has a strong antipyretic effect, although the neurochemical mechanism underlying this effect is not entirely known. However, there is evidence to suggest that it reduces fever by acting centrally on the hypothalamus’s major heat-regulating center. The central nervous system (CNS) plays a role in the antipyretic effect, which is achieved by the suppressing of prostaglandin synthesis (or one step before prostaglandin E2 generation). Malvar et al. 2011, however, showed that the hypothalamic suppression of prostaglandin E2 synthesis was not in the mechanism underlying the antipyretic activity of metamizole [30,31]. The antipyretic effect of metamizole may, instead, still be brought on by unexplained mechanisms in terms of its antinociceptive action [29,31].

### 4.3. Pharmacokinetics

**Figure 1:**
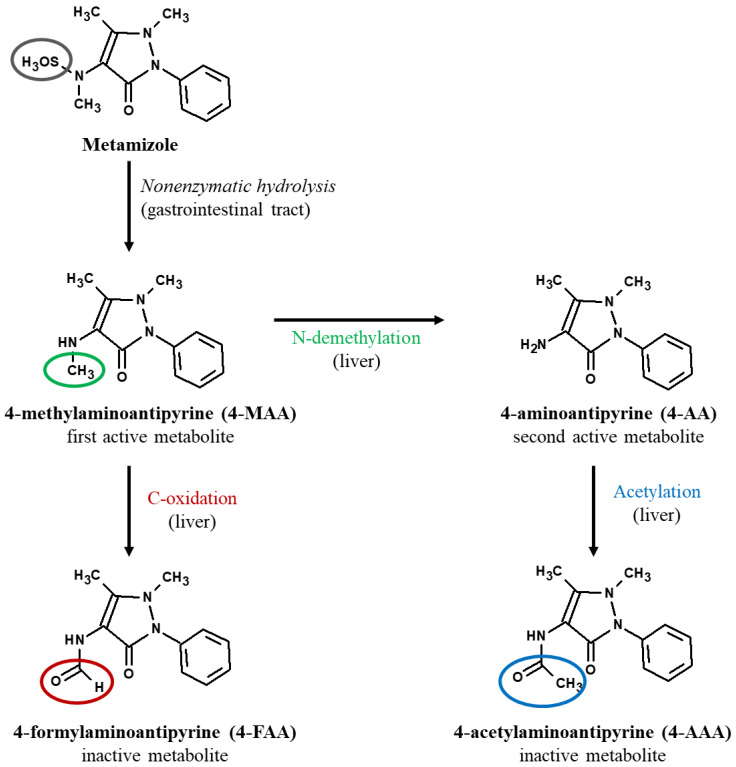
Hepatic metabolism of metamizole (adjusted after Lutz, 2019 [32]).

Metamizole is largely metabolized in the liver, and this action is mediated by the cytochrome P450 complex. Taking this into account, the hepatotoxic potential of this drug is easy to imagine [21]. Metamizole is considered a prodrug, which indicates that it metabolizes and is broken down in the body very rapidly—when it is administered orally—via a nonenzymatic hydrolysis reaction into its primary metabolite 4-methylaminoantipyrine (4-MAA). This form is completely absorbed in gastric juice and then transferred to the liver, where it is mediated by the cytochrome P450 (CYP) 3A4 system, which is essential for metabolic reactions (Figure 1). Further, 4-MAA undergoes two metabolic processes in the liver. The first reaction forms 4-aminoantipyrine (4-AA), which is a second active metabolite. The second reaction, a C-oxidation, forms a first inactive metabolite called 4-formylaminoantipyrine (4-FAA). In addition, when 4-AA is further acetylated, it then forms another inactive metabolite 4-acetylaminoantipyrine (4-AAA) [20,21,32]. 

The primary metabolite, MAA, is 50 times more active than metamizole as a COX inhibitor, while the AA metabolite is less active than metamizole. Therefore, both metabolites exhibit clinically relevant properties, such as rapid onset and long duration of effect, allowing dosing intervals of 6 to 8 h. However, the half-life of MAA is dose-dependent. The other two metabolites, FAA and AAA, are inactive. As such, the full effects of these metabolites, i.e., the metabolites that generate excellent analgesic action as well as those that cause severe side effects, are still unknown [29,31]. 

**Figure 2:**
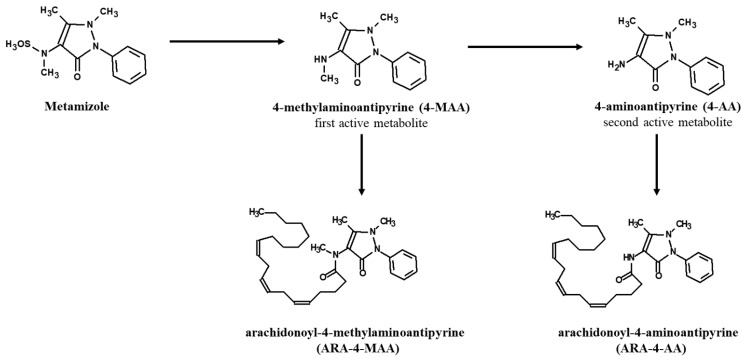
Hepatic metabolism of metamizole. Two further metamizole metabolites were discovered, representing the arachidonoyl amides of 4-MAA and 4-AA (adjusted after Lutz, 2019, [32]).

Using liquid chromatography–mass spectrometry (LC–MS), two additional metabolites, arachidonoyl-4-methylaminoantipyrine (ARA-4-MAA) and arachidonoyl-4-aminoantipyrine (ARA-4-AA), were identified to be present following oral metamizole administration, in recent research. Further metabolism of 4-MAA and 4-AA results in the production of active arachidonoyl amides, whose presence in the mouse spinal cord and brain has been established (Figure 2). The idea that these metabolites are produced in the central nervous system (CNS) is supported by the fact that fatty acid amide hydrolase (FAAH), an enzyme that is found in high concentrations in the brain, is involved in the formation of arachidonoyl amides [32,33]. 

The bioavailability of 4-MAA is 85% for tablets, 89% for drops, 54% for suppositories, and 87% for intramuscular administration. All of the important metabolites also pass into the cerebrospinal fluid. According to intravenous administration, 90% of the metabolites are excreted in the urine, and less than 10% are excreted in the feces [21]. 

Both active metabolites (4-MAA and 4-AA) have a dose-dependent half-life. For 4-MAA, when used in healthy adults, it varies between 2.5 h (for 750 mg metamizole) and 3.5 h (3000 mg metamizole), and for AA, it varies between 4 h and 5.5 h, respectively. When compared to younger people (t1/2 = 2.5 h), the elderly underwent a longer time to eliminate 4-MAA (t1/2 = 4.5 h). The duration is proportional to creatinine clearance [29,31]. 

In comparison to healthy individuals, the elimination of the 4-MAA metabolite is delayed in patients with liver disease. The plasma half-life of the 4-MAA metabolite is four times longer in people with cirrhosis of the liver compared to healthy individuals [29,31]. 

### 4.4. Use of Metamizole in Animals

From a veterinary medicine perspective, the literature does not provide conclusive data on the efficacy of this substance, but as a starting point we have a variety of studies conducted on pigs, cattle, canids, equines, as well as a meta-analysis of the therapeutic effects of dipyrone on dogs by Brazilian researchers [34]. 

Recent research has examined the pharmacokinetics of 4-MAA and 4-AA in a number of species following a single IV or IM dose of metamizole, including sheep [19], donkeys [20], pigs [35], horses [36], cats [23], dogs [37], goats [38], and calves [35,39]. 

In the veterinary medical market, metamizole is approved for use in the following species: swine, cattle, equines, and canines and has been shown to be suitable for parenteral administration in doses ranging from 25–50 mg/kg [36,37]. 

For perioperative analgesia in mice and rats, metamizole was used in 8–9% of cases, followed by paracetamol, flunixin meglumine, and butorphanol. For postoperative pain management, metamizole was the third most commonly used medication, after buprenorphine and carprofen [37,40]. 

In a study in dogs, it was confirmed that it may be a suitable substance in the management of postoperative pain, but also in oncology patients, in combination with other NSAIDs and opioids [34]. A major aspect is that by administering metamizole, adverse effects on the gastrointestinal and renal systems, due to the administration of non-steroidal anti-inflammatory drugs (NSAIDs) and the depressant effects of opioids, can be avoided.

A dose of 50 mg/kg administered every 6 h to rabbits and dogs was used for the effective treatment of pain [38,41]. 

Investigations on the long-term use of metamizole in dogs during surgical or anesthetic intervention is still required. One study showed that a dose of 25 mg/kg has been used in dogs in the postoperative period in order to supplement analgesia in patients undergoing mandibulectomy [39,42]. 

In a study conducted on cats after administration of metamizole, it was metabolized to the final and active forms after a longer time had passed, when compared to other species. However, the plasma half-life of MAA and AA metabolites was longer compared to previous studies in equine and sheep [23]. 

Metamizole’s pharmacokinetic characteristics, following intravenous and intramuscular injection, was examined in a research study on horses. The pharmacokinetic profile of the MAA metabolite was similar between the groups, while the AA metabolite was found at twice the amount after intravenous administration. Although further studies are needed to better understand metamizole’s metabolism, as well as its safety in use, the reported difference in AA concentrations may be clinically negligible in horses [36,40]. 

The minimum alveolar concentration (MAC) of sevoflurane in rats was shown to be lower after metamizole treatment. Although prolonged administration of metamizole may be associated with hyperalgesia and tolerance, which can modify sevoflurane MAC in rats, a 20% reduction in MAC has still been determined after a low dose of metamizole (15 mg/kg), compared to a 30% reduction after administration of a higher dose (300 mg/kg) [41].

In calves undergoing umbilical surgery, a single preoperative administration of metamizole was used for analgesia; further, it has been recommended to be used in combination with xylazine, ketamine, isoflurane, and meloxicam [42]. 

As is the case in all other species, further studies are needed to assess the effect of metamizole for the purposes of analgesia and safety.

## 5. Conclusions

Metamizole is still a targeted product, in certain circumstances, due to its link with agranulocytosis. However, on the other hand, it is still a commonly used product in certain countries. Future randomized clinical trials are needed in order to determine the safety and efficacy in use, as the benefits of using metamizole should be weighed together with the risks, especially when compared to other NSAIDs [21]. 

Due to its weak anti-inflammatory properties, it is used in cases where there is a need to confer analgesia, but without the inducement of anti-inflammatory properties. There are many studies that have compared the analgesic effect of metamizole with the analgesic effect of other substances. A single dose of metamizole has been shown to have an analgesic effect similar to substances more commonly used in the therapy of moderate to severe pain in postoperative management [21]. 

An advantage of metamizole administered with other opioids is that it lowers the dose required of the opioid that is needed in order to achieve the desired analgesia. Pain is a very common problem among patients with cancer, with a 90% prevalence in advanced cancer. Further, NSAIDs and opioids have been shown to have additive (synergistic) effects in animals, including metamizole. Metamizole, however, does have a synergistic effect together with morphine in postoperative pain, and has been used for many years as a substitute for other opioid analgesics [22]. 

## Data Availability

Not applicable.
